# Targeting Hepatitis B Virus With CRISPR/Cas9

**DOI:** 10.1038/mtna.2014.68

**Published:** 2014-12-16

**Authors:** Christoph Seeger, Ji A Sohn

**Affiliations:** 1 Institute for Cancer Research, Fox Chase Cancer Center, Philadelphia, Pennsylvania, USA

**Keywords:** antiviral therapy, chronic hepatitis B, covalently closed circular DNA, CRISPR/Cas9, hepatitis B virus, nonhomologous end joining

## Abstract

Hepatitis B virus persistence in infected hepatocytes is due to the presence of covalently closed circular DNA (cccDNA), the template for the transcription of viral RNAs. Antiviral therapies with nucleoside analogues inhibit replication of HBV DNA in capsids present in the cytoplasm of infected cells, but do not reduce or destroy nuclear cccDNA. To investigate whether cccDNA derived from infectious HBV could be directly targeted for destruction, we used the CRISPR/Cas9 system in HepG2 cells expressing the HBV receptor sodium taurocholate cotransporting polypeptide (NTCP). We tested different HBV-specific guide RNAs and demonstrated that they could inhibit HBV infections up to eightfold. Inhibition was due to mutations and deletions in cccDNA similar to those observed with chromosomal DNA cleaved by Cas9 and repaired by nonhomologous end joining (NHEJ). Interferon alpha (IFN-α) did not have a measurable effect on the antiviral activity of the CRISPR/Cas9 system, suggesting that Cas9 and NHEJ activities are not affected by induction of an innate immune response with the cytokine. Taken together, our results demonstrated that Cas9 can be recruited to cccDNA, opening the possibility for the development of future antiviral strategies aimed at targeting cccDNA for endonucleolytic cleavage with small molecules.

## Introduction

**Figure 1:**
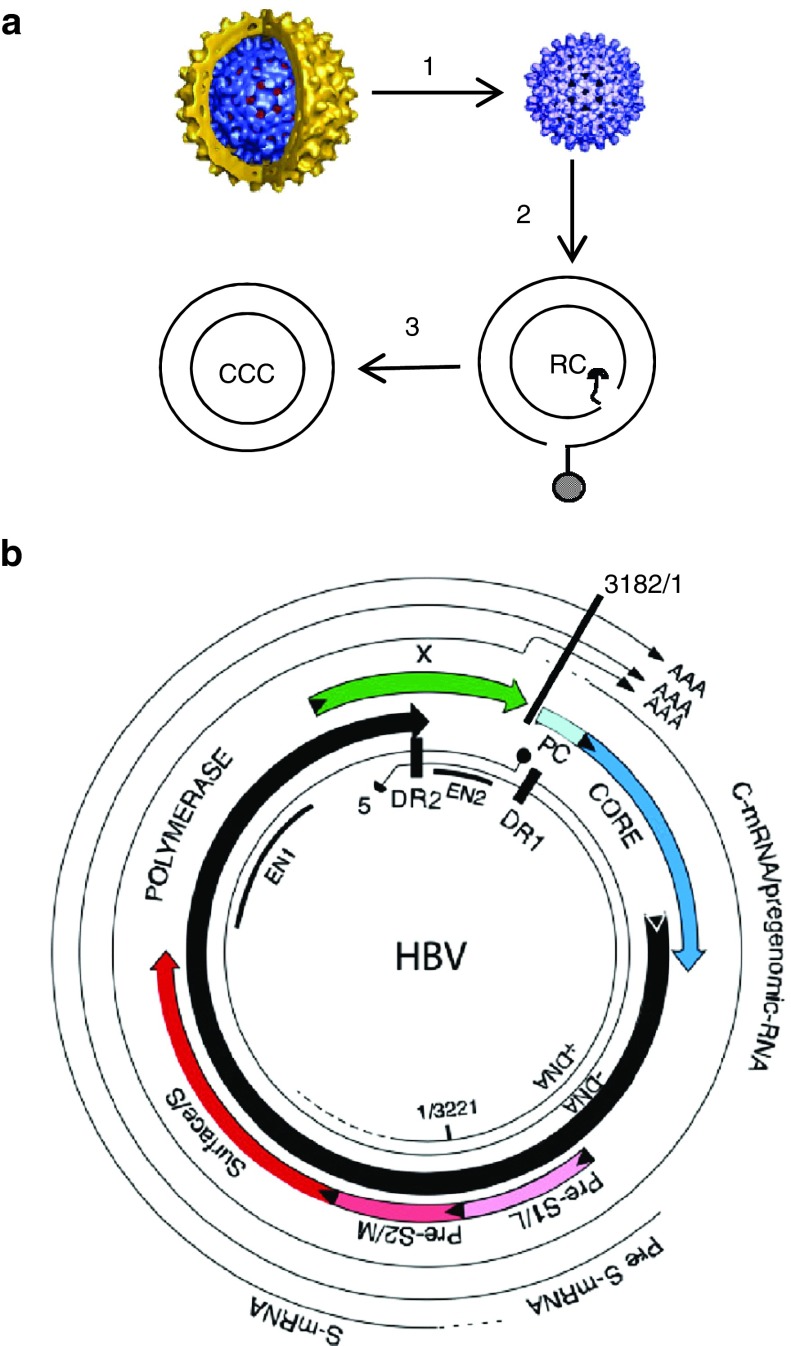
**Life cycle and genomic organization of HBV.** (**a**) The enveloped infectious HBV particle is uncoated after infection releasing the capsid (1) that migrates to the cell nucleus where it delivers the genomic rcDNA (2) which is converted into cccDNA (3), the template for transcription of the viral RNAs. (**b**) Physical map of HBV with the four open-reading frames and viral RNAs. Also indicated are the positions of enhancers I and II (EN1, EN2). For additional details, see ref. 5.

Over 350 million people worldwide are infected with hepatitis B virus (HBV).^1^ About 10% of carriers suffer from chronic hepatitis B (CHB), a condition that can lead to cirrhosis and hepatocellular carcinoma. It is estimated that over 700 thousand HBV-infected patients succumb to the sequelae of infection each year.^2^ Antiviral therapies developed during the past 20 years are effective in suppressing, but not eliminating, HBV infections.^3^ The apparent lack of a sustained viral response following cessation of antiviral therapy is due to the stability of the covalently closed circular (ccc) DNA.^4,5^ CccDNA is formed from the relaxed circular (rc) genomic DNA by a DNA repair mechanism that is still not understood (**Figure 1**).^6^ Once established as a minichromosome, cccDNA functions as a template for the synthesis of four RNA transcripts, pregenomic (pg) RNA, preS, S, and X RNAs.^4,7^ DNA replication occurs in core particles present in the cytoplasm of infected cells by reverse transcription of pg RNA.^8,9^ DNA containing core particles assemble with envelope proteins, enter the secretory pathway, and are secreted into the blood stream. Nucleoside analogue-based antiviral therapies inhibit the viral reverse transcriptase, and hence the formation of progeny virus. However, they do not target nuclear cccDNA in infected hepatocytes. What makes matters worse is that infected hepatocytes harbor multiple copies of cccDNA as a consequence of an intracellular DNA amplification mechanism.^10^ CccDNA amplification occurs during the initial phase of an infection when levels of viral envelope proteins in hepatocytes are still low, favoring retrograde passage of core particles into the cell nucleus over assembly with envelope proteins.^11^

A key issue for antiviral therapy is the development of strategies that can either alone or in combination with nucleoside analogues lead to the reduction and eventual eradication of cccDNA from infected hepatocytes. Information about the stability of cccDNA has been forthcoming from studies with cell culture models for viral infection and replication as well as from the analysis of viral DNA extracted from liver biopsies of animals recovering from transient infections. Whereas in nondividing primary hepatocyte cultures cccDNA is stable,^12,13^ in dividing tissue culture cells, cccDNA is passed on to daughter cells suggesting that it can “survive” mitosis.^14,15^ Results from experimentally infected chimpanzees and woodchucks indicated that during resolution of acute infections loss of cccDNA occurs primarily by killing of infected heaptocytes by cytotoxic T cells and possibly by noncytolytic mechanisms induced by cytokines and other unknown factors that are part of the immune-mediated clearance phase.^16,17,18,19,20^ The exact level of contribution of each event to the decline of cccDNA levels observed during the resolution of an infection is still not resolved. A major gap in knowledge concerns the nature of the host factors that play a role, if any, during noncytolytic clearance of cccDNA. A recent report claimed that interferon alpha (IFN-α and lymphotoxin beta (LT-β) pathways might cause the destruction of cccDNA by certain endonucleases following deamination of the minus strand DNA by APOBEC3A (A3A) and A3B.^21^ However, whether these results bear any relevance for clearance of HBV infections during acute infections remains to be determined.^22^

Efforts to develop strategies that could induce cccDNA loss during CHB have been hampered by the lack of tissue culture systems that are permissive for HBV infection and at the same time are amenable to experimental manipulations, such as gene knockout technologies. However, identification of the HBV receptor, sodium taurocholate co-transporting polypeptide (NTCP), opened the possibility for establishment of HepG2 cell lines permissive for infections with HBV produced in tissue culture cells.^23,24^ In this report, we demonstrate the utility of this system for investigations on cccDNA formation and stability by combining it with the clustered regularly interspaced short palindromic repeats (CRISPR)/Cas9 platform for efficient gene knockout.^25,26,27^ Using this system, we investigated whether cccDNA formed from infectious virus can be targeted by a recombinant endonuclease and whether cellular enzymes can either repair or destroy cccDNA under selected conditions.

## Results

### Production of NTCP/Cas9 cells permissive for HBV infection

**Figure 2:**
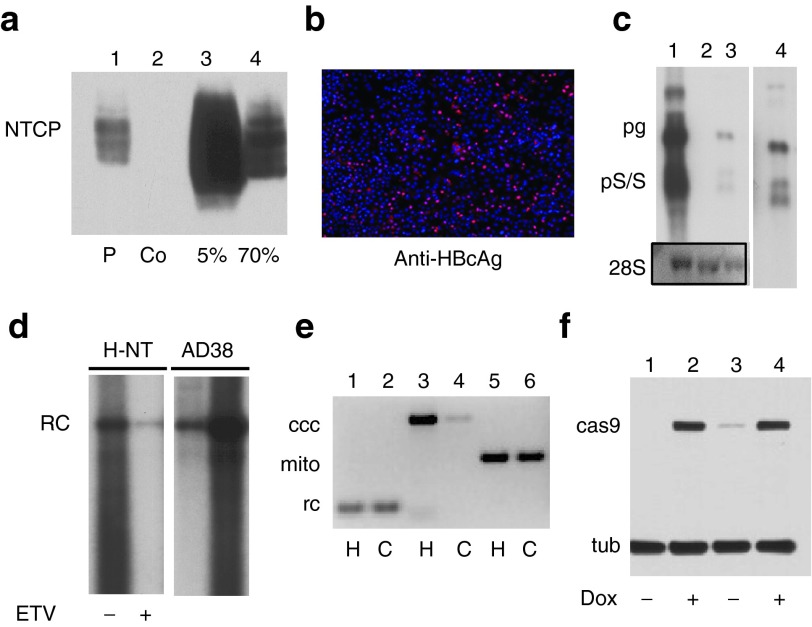
**HBV infection of NTCP/Cas9 cells.** (**a**) Expression of NTCP in HepG2 cells expressing Cas9. Cells were sorted by FACS to enrich for populations with high NTCP levels. Lane 1, cell pool prior to FACS; lane 2, control HepG2 cells; lanes 3 and 4, 5 and 70% fraction of FACS sorted cells with the highest GFP expression levels. (**b**) Infection of NTCP/Cas9 cells with HBV derived from HepAD38 cells. Cells were processed for IF analysis with HBcAg-specific antibody C1-5 8 days after infection. (**c**) Northern blot analysis of total RNA extracted from HepG2/NTCP cells infected with HBV (lane 3) and HepAD38 cells as a control (lane1). Uninfected cells (lane 2) and ribosomal 28S RNA served as a control for the amount of RNA loaded onto each well. Lane 4 is a longer exposure of lane 3. (**d**) Analysis of core DNA extracted from HepG2/NTCP (H-NT) cells infected with HBV. DNA was extracted 14 days after HBV infection (lanes 1 and 2). Cells were cultured without (lane 1) and with entecavir (ETV, 10 µg/ml)) (lane 2). DNA extracted from HepAD38 (AD38) cells served as a control (lanes 3 and 4). Lane 3 was loaded with 1/10th of the amount of DNA used for lane 2. (**e**) PCR analysis of HBV DNA extracted from infected NTCP/Cas9 cells. DNA was extracted with the Hirt procedure (H) from total cell extracts or from the cytoplasm (C) of the HBV-infected cells. Primers used for the PCR reactions are described in **Supplementary Table S1**. Ccc, CCC DNA–specific primers overlapping the cohesive overlap in rcDNA (**Figure 1a**); mito, mitochondrial DNA-specific primers; rc, primers for amplification of rcDNA. (**f**) Conditional expression of Cas9 in two different cell clones. Cells were cultured with (lanes 2 and 4) and without (lanes 1 and 3) dox. dox, doxycycline tub; tubulin.

In a first step, we established HepG2 cells expressing NTCP from a lentivirus vector, NT-GFP, that also expresses GFP (Materials and Methods). HepG2/NTCP cells can be enriched by fluorescence activated cell sorting (FACS) for subpopulations that express high levels of the receptor. We found that permissiveness for HBV infections directly correlated with NTCP levels in these cells (**Figure 2a**). The fraction of HepG2/NTCP cells that could be infected with HBV derived from the supernatant of HepAD38 cells,^28^ reached up to 50–60%, primarily depending on NTCP cells expression levels (**Figure 2b**). HBV-infected HepG2/NTCP expressed the three major viral RNA transcripts, pregenomic RNA and preS and S RNAs (**Figure 2c**) as well as cytoplasmic HBV DNA that is characteristic of hepadnavirus replication (**Figure 2d**). As expected, accumulation of cytoplasmic DNA was reduced when cells were maintained in the presence of entecavir, an HBV reverse transcriptase inhibitor (**Figure 2d**). Southern blot analysis was not sensitive enough to detect cccDNA, suggesting that the copy number of cccDNA in infected cells was very low, most likely not exceeding one. However, cccDNA was detected in infected HepG2/NTCP cells by PCR using primers that favor amplification of cccDNA over the more abundant rcDNA (**Figure 2e**). To combine the HBV infection system with the CRISPR/Cas9 platform for efficient gene inactivation, we introduced in a second step the endonuclease Cas9 into HepG2/NTCP cells with a second lentivirus vector, pCW-cas9.^27^ Cas9 expression is under the control of the CMV-tetracycline (tet) promoter and can be induced with doxycycline (dox) (**Figure 2f**).

### CRISPR/Cas9 targeting of HBV cccDNA

To investigate whether cccDNA derived from input HBV can be targeted for cleavage and destruction by Cas9, we designed several sgRNAs spanning the ENII/CP region and the PC regions on the HBV genome (**Figure 1b** and **Table 1**). We selected this region because it encodes HBx and the overlapping control region, both important for transcription of pregenomic RNA encoding the HBV core antigen (HBcAg), which can be detected in infected cells by immunofluorescence with available antibodies. We validated the cleavage efficiency of the sgRNAs in HepAD38/Cas9 cells carrying an integrated genome derived from HBV genotype ayw.^28^ Infection of HepAD38/Cas9 cells with lentiviruses expressing the relevant sgRNAs resulted in cleavage of integrated HBV DNA as determined with the Surveyor assay for the detection of mismatched DNA in PCR amplified DNA fragments^29^ (**Supplementary Figure S2**). The results showed that all five sgRNAs tested efficiently cleaved chromosomal HBV DNA.

**Figure 3:**
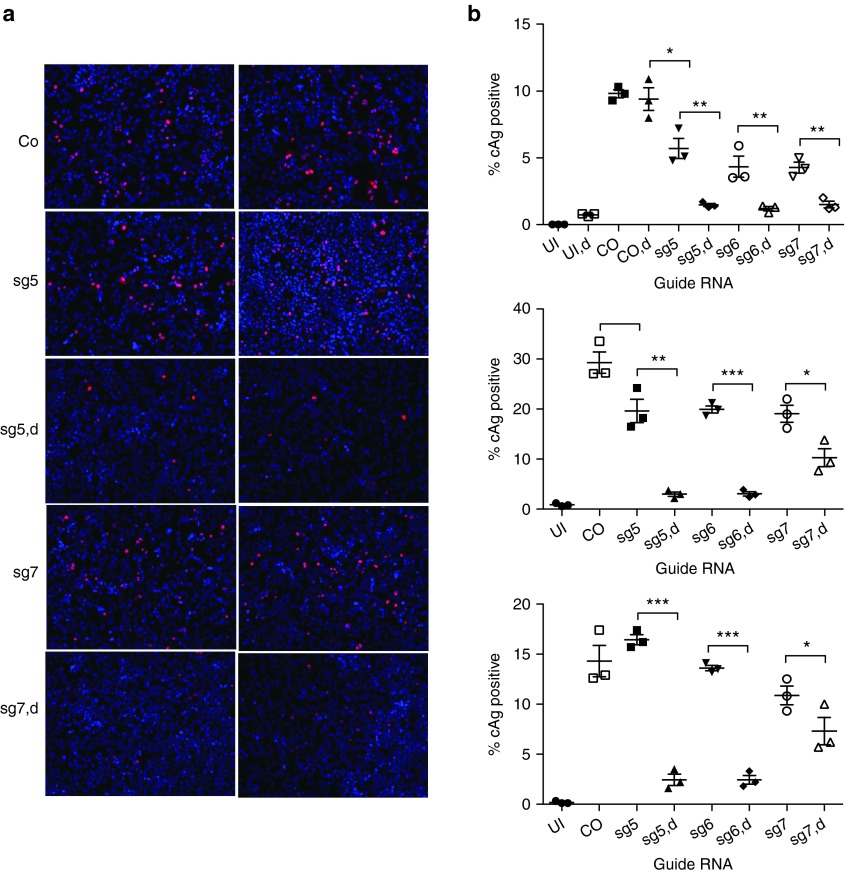
**Inhibition of HBcAg expression with CRISPR/Cas9.** (**a**) The results from an HBV infection on NTCP/cas9 cells expressing sg5 and sg7 guide RNAs. d, doxycycline. Immunofluorescence was performed with HBcAg-specific antibody C1-5 8 days after HBV infection. (**b**) Results from a quantitative analysis of HBcAg-positive cells from three different experiments performed with sgRNAs 5, 6, and 7, as described in **a**. Co, control; UI, uninfected. *P < 0.05; **P < 0.01; ***P < 0.001.

To determine the potential antiviral activity of CRISPR/Cas9 against HBV, we infected NTCP/Cas9 cells first with lentiviruses expressing individual sgRNAs and then with HBV. HBV infected cells were maintained in medium containing dox to induce Cas9 expression. The fraction of HBV-infected cells was determined by immunofluorescence with an HBcAg antibody 7–10 days post HBV infection (**Figure 3a**,**b**). The results showed that expression of sg5 and sg6 guide RNAs inhibited HBcAg expression between 6- and 10-fold compared to controls that either were not infected with the respective lentivirus vector or were maintained in the absence of dox to prevent Cas9 expression. Sg7 exhibited a weaker antiviral effect reducing the fraction of HBcAg-positive cells between twofold and sixfold. In two experiments, we observed a slight reduction in the fraction of HBV-infected cells in lentivirus-infected cells maintained without dox compared with uninfected control cells, which could be attributed to leakiness of the tetracycline promoter resulting in low level expression of Cas9 even in the absence of dox (**Figure 3b**). Alternatively, we cannot rule out that small differences in expression of NTCP existed between control cells and cells expressing different sgRNAs. This would also explain higher infection levels observed with cells expressing sg5 compared with other cells in the experiment (**Figure 3b**).

### Analysis cccDNA in HBV-infected NTCP/Cas9 cells

**Figure 4:**
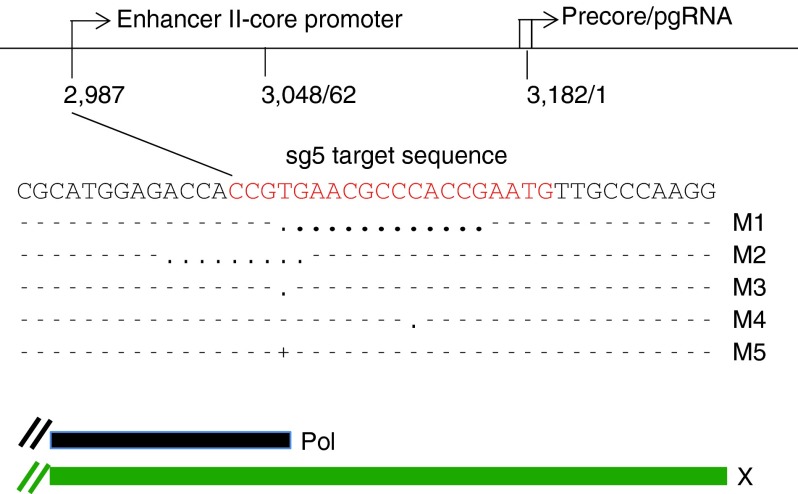
**Nucleotide sequence analysis of cloned cccDNA.** (**a**) The target sequence (plus strand) of sg5 guide RNA on the HBV genome and the position of five selected mutations identified in cloned PCR fragments derived from cccDNA of HBV-infected cells expressing sg5 are inidcated. Deletions are indicated with dots, insertions with a plus sign. Position 2,987 corresponds to the 3′ end of sgRNA 5 (sg5) abutting the PAM sequence (GTT). For the positions of the pol and X genes on the HBV genome, see **Figure 1b**.

So far, our results indicated that HBV infections can be inhibited with the CRISPR/Cas9 system, implying that newly formed cccDNA is cleaved and either repaired by NHEJ, like chromosomal DNA, or destroyed by cellular nucleases. To determine the fate of cccDNA, we extracted DNA from HBV-infected NTCP/Cas9 cells expressing sgRNAs 5, 6, and 10 under conditions described in the previous section. Sg10 RNA exhibited an antiviral activity similar to that of sg5 and sg6 (results not shown). Purified DNA was PCR amplified with cccDNA-specific primers (**Supplementary Table S1**, **Figure 2e**), and the PCR products were cloned and sequenced. As a control, we analyzed DNA from HBV-infected cells (maintained in dox-free medium) expressing sgRNAs in the absence of Cas9 expression. As expected, all (26) sequenced clones were identical with wild type (**Table 2**). In contrast, clones derived from cells expressing sgRNAs and Cas9 exhibited mutations characteristic of Cas9 cleavage (**Tables 2** and **3**). The most frequent type of mutation was a single-nucleotide insertion or deletion within the sequence motif corresponding to the respective sgRNA (**Figure 4**). In addition, we observed deletions spanning 2–30 nucleotides in length as well as larger deletions spanning up to 2.3 kb in length. In about two-thirds of the larger deletions, one of the two break points mapped to the Cas9 cleavage site near the PAM (protospacer adjacent region) sequence abutting the 3′ end of the sgRNA.

**Figure 5:**
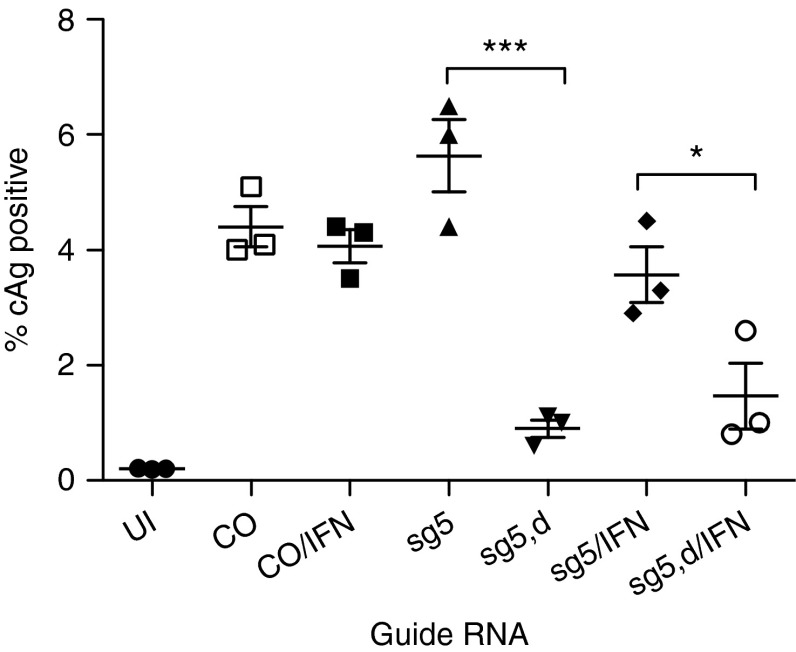
**Effect of IFN-α on Cas9 cleavage of cccDNA.** Results from a quantitative analysis of HBcAg-positive cells from an experiment performed with sgRNA 5 with and without IFN-α as in **Figure 3** is shown.

A comparison between the results obtained with the immunofluorescence (IF) IF assay (**Figure 3**), and the sequence analysis (**Table 2**) revealed a good correlation between the fraction of HBcAg-positive cells and the fraction of wild-type clones for sg5 RNA. Sg5 RNA reduced the fraction of HBcAg-positive cells about sevenfold, indicating that about 14% of the infected cells expressed wild-type genomes (experiment 3 in **Figure 3**), which is consistent with the presence of about 18% wild-type clones observed with the sequence analysis of the cloned PCR fragments. In a different experiment where sg5 RNA was expressed in cells treated with IFN-α (**Figure 5**, described below), the fraction of infected cells was reduced only 2.5-fold, which correlated with an increase in the fraction of wild-type clones to 33%. In contrast, the same correlation was not as pronounced with sg6 RNA, which reduced the fraction of HBcAg-positive cells fivefold to sixfold, while the fraction of wild-type clones reached 45%, more than twice the expected value. While the reason for this discrepancy is not known, it is possible that sg6 induced larger deletions that could not be PCR amplified from cccDNA.

To determine whether IFN-α could enhance the antiviral activity exhibited by the CRISPR/Cas9 system, we treated NTCP/Cas9 cells expressing sg5 guide RNA 5 days after HBV infection for 72 hours with the cytokine (2,000 IU/ml). Under those conditions, IFN-α did not significantly reduce the fraction of HBcAg-positive cells when Cas9 was expressed (**Figure 5**). Similarly, we did not observe an increase in the fraction of mutations determined from the cccDNA analysis (**Table 2**), nor did we detect any additional types of mutations. However, IFN-α caused cell death at the high concentrations used in our experiments (2,000 and 1,000 IU/ml), in particular when cells were treated for 5 or 8 days with the cytokine (**Supplementary Figure S3**). Toxicity was even more apparent when the HBV-infected cells were treated with dox to induce Cas9.

## Discussion

We have developed a CRISPR/Cas9 platform for efficient inactivation of viral genes in NTCP expressing HepG2 cells permissive for HBV infection. We demonstrated that cccDNA can be targeted and inactivated as a consequence of Cas9-induced double-strand DNA breaks. While several previous reports demonstrated that transfected plasmid DNA corresponding to the HBV genome can be targeted by zinc fingers or transcription activator-like effector nucleases (TALENs), this is the first report to demonstrate that cccDNA formed from infectious virus can be cleaved and repaired like chromosomal DNA.^30,31,32^ Although not demonstrated experimentally in this report, this system can also be used for inactivation of cellular genes, which will enable future investigations on the role of host genes in the HBV life cycle.

While our results revealed that cccDNA derived from infectious virus is indeed accessible for Cas9 enzymatic cleavage, they also demonstrated that cleaved cccDNA is rapidly repaired, most likely by the NHEJ pathway normally used for repair of double-strand DNA breaks.

In this context, it is interesting to consider a previous report describing formation of a cccDNA species of the HBV-related duck hepatitis B virus from a double-stranded linear precursor.^33^ Such precursors are formed as a consequence of a so-called *in situ* priming reaction of plus strand DNA synthesis.^34^ While the nucleotide sequence of the joint DNA strands was not examined, DNA repair was dependent on Ku80 implying that NHEJ played a role as observed in our study with cccDNA derived from HBV rcDNA.

The nature of CRISPR/Cas9 mutations in cccDNA is similar to those observed with chromosomal DNA.^35^ The most frequent mutations (66%) were single-nucleotide deletions or insertions, followed by deletions that spanned over 100 nucleotides (19%) and smaller deletions of less than 30 nucleotides in length (12%). The reason for the vast difference in the size of the deletions spanning a few nucleotides to more than 2 kb is not known. One possibility might be that some cccDNA molecules are cleaved twice due to an off-target cleavage in addition to the target-specific event.^36,37^ However, considering that we observed large deletions with three different sgRNAs examined suggest that this is an unlikely explanation. However, it appears that sequences between positions 7 and 12 near DR1are preferred targets for the second cleavage site observed with sg5 and sg6 guide RNAs, respectively (**Table 3** and **Figure 1b**). Perhaps, in rare cases, Cas9 cleavage might occur prior to cccDNA formation on rcDNA, leading to ligation of the 3′ end of minus strand DNA to the 5′ end of the cas9 cleaved minus strand DNA.

With sg5 RNA, we found a very good correlation between the sgRNA-induced inhibition of HBcAg expression and the ratio of wild-type and mutated clones obtained from PCR-amplified cccDNA (**Figure 3** and **Table 2**). A described above, the most frequent mutations observed were single-nucleotide insertions and deletions that resulted in truncation of HBx and also in a change in the overlapping ENII/CP sequence motif (**Figure 4**). Therefore, it is not possible to determine the cause for the observed inhibition of HBcAg expression with certainty. We favor the conclusion that impairment of HBx expression is the primary cause for this inhibition, because previous studies have demonstrated that HBx activity is required for transcription of preC/C RNA from episomal cccDNA templates.^38,39,40^ Moreover, we consider it unlikely that different single-nucleotide deletions or insertions would significantly abrogate the activity of the ENII/PC regulator sequence. However, proof for this conclusion would require demonstration that the mutants can be rescued with wild-type HBx *in trans*. Attempts to transfect HBV-infected NTCP/Cas9 cells with an HBx expression plasmid have failed so far, because the DNA transfection efficiency was too low to detect a possible rescue of HBcAg expression. Efforts to improve the conditions for transfection are in progress.

Because a goal of this study was to investigate the stability of cccDNA following endonucleolytic cleavage, we also investigated whether activation of an antiviral innate immune response could augment the antiviral effect of the CRISPR/Cas9 system in conjunction with NHEJ. In an initial effort, we tested whether treatment of cells with IFN-α could further reduce the fraction of HBcAg-positive cells following HBV infection and sgRNA expression. These experiments were also motivated by a recent report claiming that IFN-α and tumor necrosis factor α could activate APOBEC 3A and B leading to enzymatic digestion of cccDNA.^21^ In contrast to the previous report, where HepaRG and primary hepatocyte cultures cells were treated for 10 days or more with the cytokines, in our experiments, IFN-α was added to HepG2 cells for only 3 days before cells were analyzed for HBcAg expression or harvested for cccDNA analysis. Moreover, we did not use 3D-PCR, a nested PCR reaction designed to selectively amplify DNA with a high A/T content, which can be the result of APOBEC-mediated deamination of C residues in rc or cccDNA.^21,41^ Nevertheless, under our selected conditions, we did not obtain any evidence for an enhanced antiviral activity against HBV in the presence or absence of sgRNAs. Moreover, we did not detect differences in the nature of the mutations in PCR clones derived from cccDNA. We observed some toxicity associated with IFN-α treatment and induction of Cas9 in HBV-infected NTCP/Cas9 cells (**Supplementary Figure S3**). It might be due to the combination of HBV infection with the CRISPR/Cas9 platform. Additional experiments will be required to investigate the activities of IFNs and other cytokines in this system. Finally, it might be possible to identify sgRNAs targeting additional regions on the HBV genome that might inactivate cccDNA with a significantly higher efficiency than we observed so far with our experiments.

While our results suggest that mutated cccDNA, even with substantial deletions, is relatively stable in HepG2 cells during the 7–10-day observation period used in our studies, they do not preclude the possibility that mutated cccDNA is lost more rapidly than wild-type DNA over longer time intervals spanning weeks and even months. Moreover, additional information about the exact timing of cccDNA formation and subsequent cleavage by Cas9 might help to obtain better estimates for the half-lives of mutated cccDNA. Whatever, the stability of mutated cccDNA might be, the fact that it is functionally inactivated as a consequence of the NHEJ emphasizes the potential of this repair pathway for an antiviral strategy. Although this study provided encouraging results toward a major goal in antiviral therapy against CHB, which is the therapeutic elimination of cccDNA from the liver, it leads to the more challenging issue concerning utility in the clinical setting. We favor a strategy based on small molecules that can either recruit a cellular endonuclease to cccDNA leading to repair by NHEJ. Such a strategy would require identification of a chemical structure that can recognize a specific motif on the cccDNA minichromosome. Perhaps subtle changes in histone spacing or a combination of host and virus proteins that bind to cccDNA can be exploited to achieve this goal.

## Materials and methods

*Cells.* HepG2/NTCP cells were produced by infection of HepG2 cells with lentivirus NT-GFP; GFP expressing cells were enriched with the help of a FACS. NTCP/Cas9 cells were derived from HepG2/NTCP cells following infection with lentivirus vector pCW-Cas9^27^ and selection with puromycin (1 µg/ml).

*Antibodies.* Antibodies against the myc-tags and FLAG tags were purchased from Cell Signaling Technology (Danvers, MA) and Sigma-Aldrich (St.Louis, MO), respectively. HBcAg antibody C1-5 was obtained from Santa Cruz Biotechnology (Dallas, Tx).

*Vectors.* For the construction of lentivirus vector NT-GFP, the NTCP gene obtained from Origene (Rockville, MD) was transferred into lentivirus vector pLVX-IRES-ZsGreen (Clontech, Mountain View, Ca). For the construction of pLX-SG1, a *Nde*I–*Age*I fragment in pLX-AAVS1 sgRNA^27^ was replaced with a *Nde*I–*Age*I fragment carrying two *Bsm*BI/*Esp*3L restriction sites for cloning of guide RNA sequences with DNA oligomers (**Supplementary Table S1**). The nucleotide sequence of the *Nde*I–*Age*I fragment in pLX-SG1 is shown in **Supplementary Figure S1**. sgRNA selection was based on algorithms developed by the Zhang group (crispr.mit.edu).

*Virus and infections.* HBV was concentrated 100-fold from the culture medium of HepAD38 cells with 6% polyethylene glycol and resuspended in serum-free Dulbecco's modified Eagle medium (DMEM)/F12 medium. Infection of HepG2 cells occurred in the presence of 4% polyethylene glycol in complete serum-free medium (DMEM/F12, pyruvate, nonessential amino acids, and penicillin/streptomycin) containing 2% dimethyl sulfoxide (DMSO) with an estimated 50 genome equivalents per cell. Medium was replaced with complete medium containing 10% fetal calf serum and 2% DMSO 24 hours after infection. Cultures were incubated for 5–10 days.

*Isolation of cccDNA and rcDNA.* HBV cccDNA was isolated from infected cells by the Hirt procedure.^42^ Briefly, cells from a well of a 12-well plate were lysed in 0.45 ml of sodium dodecyl sulfate (SDS) lysis buffer (50 mmol/l Tris-HCl pH7.5, 10 mmol/l EDTA, 0.5% SDS) for 10 minutes at 37 °C. One hundred microliter of a solution of 2.5 mol/l KCl (0.5 mol/l KCl final) was added and incubated at room temperature for 30 minutes to precipitate proteins and chromosomal DNA. After centrifugation at 10,000×*g* for 10 minutes, the Hirt supernatant was extracted twice with phenol and once with butanol:isopropanol (7:3). DNA was precipitated with two volumes of ethanol at room temperature for 2 hours and resuspended in 30 µl TE (10mmol/l Tris-HCl, 1 mmol/l EDTA). To increase the efficiency of the PCR reaction, the isolated cccDNA was linearized with *EcoR*I.

HBV rcDNA was isolated from the cytoplasm of HBV-infected cells as described by Yang *et al*.^43^ Briefly, cells from a well of a 12-well plate were lysed in 0.5 ml core lysis buffer (50 mmol/l Tris-HCl, pH7.5, 1 mmol/l EDTA, 1% NP40) for 10 minutes on ice. After removal of cell debris and nuclei by centrifugation, the cleared supernatant was incubated in a buffer containing 0.5% SDS, 10 mmol/l EDTA, 100 mmol/l NaCl, and 400 µg/ml pronase for 1 hour at 37 °C. DNA was extracted with phenol and butanol:isopropanol and then precipitated with ethanol at −20 °C for 2 hours. After centrifugation, the pellet containing core DNA was resuspended in 30 µl TE.

*Immunofluorescence.* For immunostaining, cells were fixed in 96-well plates with 4% paraformaldehyde for 10 minutes and processed for immunofluorescence with HBcAg monoclonal antibody C1-5 (Santa Cruz Biotechnology, Dallas, TX). The fraction of HBcAg-positive cells was determined with an ImageXpress Micro automated microscope (Molecular Devices Sunnyvale, Ca). Images from 16 preset positions at ×10 magnification with two channels (DAPI, Cy5) were collected from each 96 wells. Images were analyzed with MetaXpress imaging and analysis software using the multi-wavelength cell scoring module.

*Statistical analysis.* Unpaired, one-tailed *t*-tests (Prism 5) were used for the statistical analyses of the results shown in **Figures 3b** and **5**.

**SUPPLEMENTARY MATERIAL** **Figure S1.** Nucleotide sequence of NdeI-to-AgeI fragment in pLX-SG1. **Figure S2.** HepAD38 cells (Ladner et al. 1997) were infected with lentivirus vectors expressing the respective guide RNAs. **Figure S3.** Toxicity of IFN-α. **Table S1.** Nucleotide sequence of DNA oligomers.

## Figures and Tables

**Table 1:**
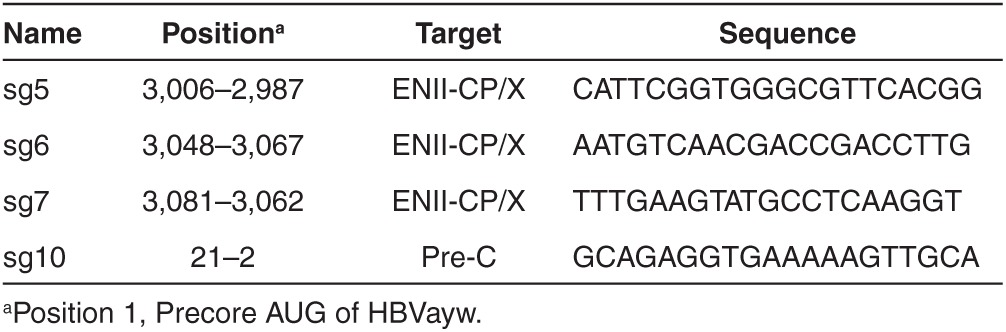
Position and nucleotide sequences of Cas9 targets on HBV

**Table 2:**
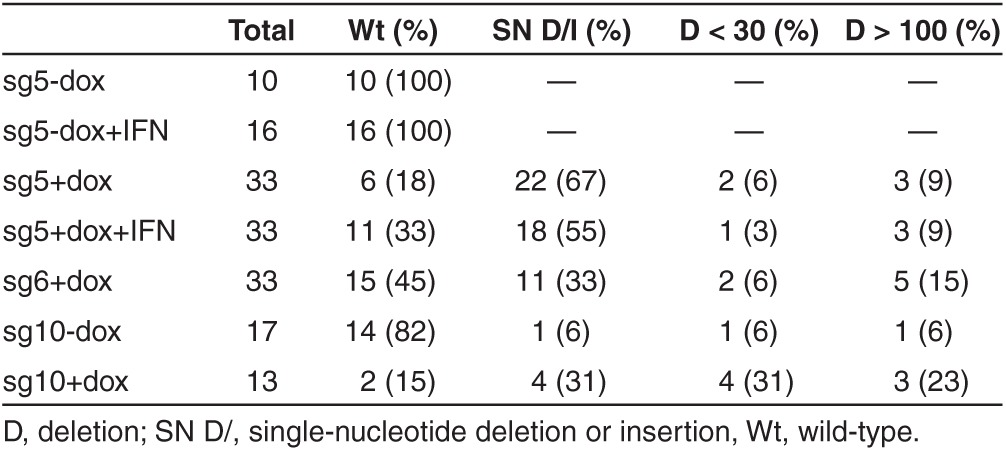
CRISPR/Cas9-induced mutations

**Table 3:**
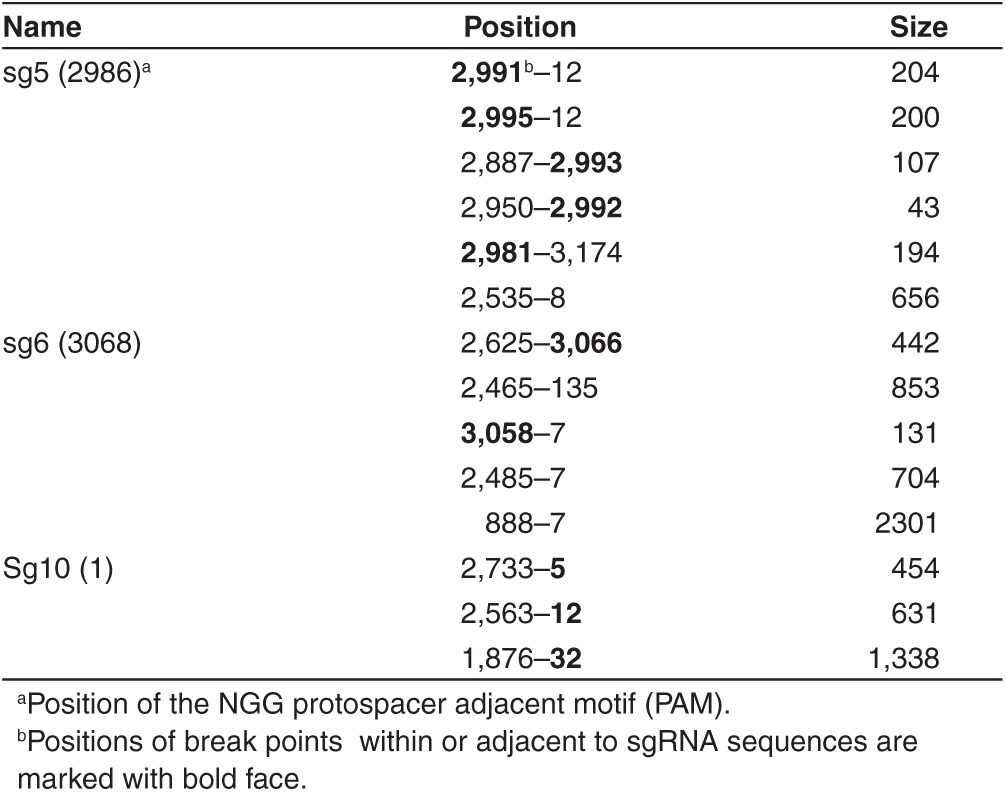
Positions of large deletions in cccDNA

## Supplementary Material

Supplementary Figure S1 Nucleotide sequence of NdeI-to-AgeI fragment in pLX-SG1.

Supplementary Figure S2 HepAD38 cells (Ladner et al. 1997) were infected with lentivirus vectors expressing the respective guide RNAs.

Supplementary Figure S3 Toxicity of IFN-α.

Supplementary Table S1 Nucleotide sequence of DNA oligomers.
