# Supplementary figures and images for: Targeting Hepatitis B Virus With CRISPR/Cas9
Source: Mol Ther Nucleic Acids. 2014 Dec 16;3(12):e216–. doi: 10.1038/mtna.2014.68 (PMC4272409; doi:10.1038/mtna.2014.68)

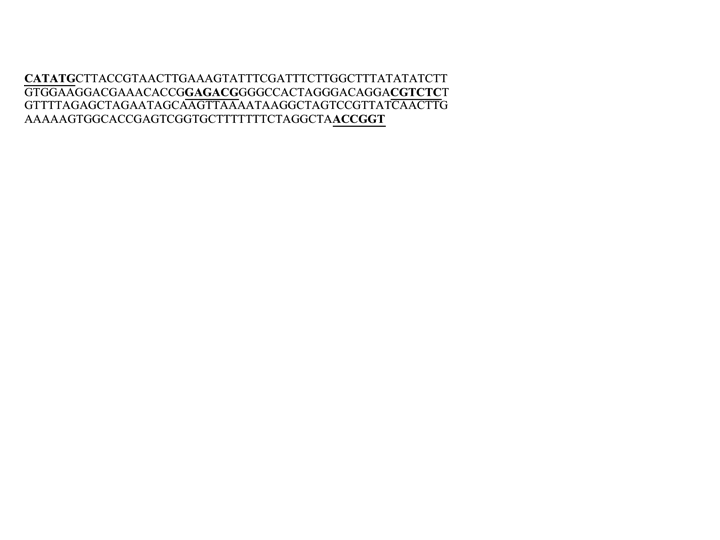

Supplement: Supplementary Figure S1 — Nucleotide sequence of NdeI-to-AgeI fragment in pLX-SG1. [file mtna201468x1.tiff]

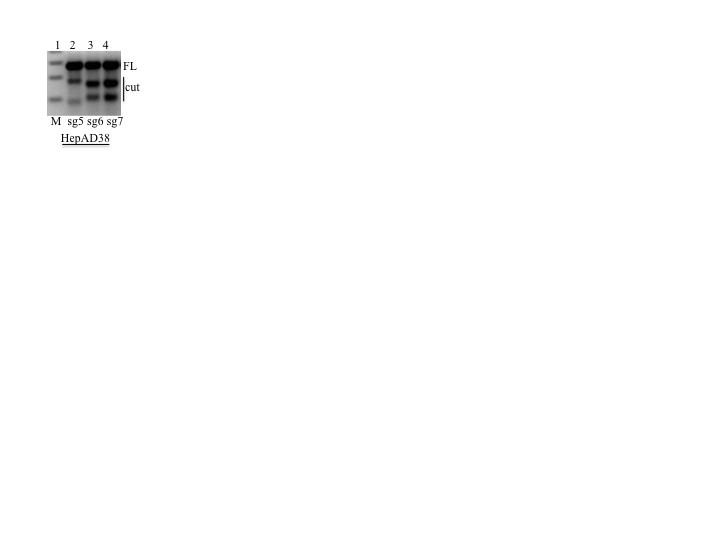

Supplement: Supplementary Figure S2 — HepAD38 cells (Ladner et al. 1997) were infected with lentivirus vectors expressing the respective guide RNAs. [file mtna201468x2.tiff]

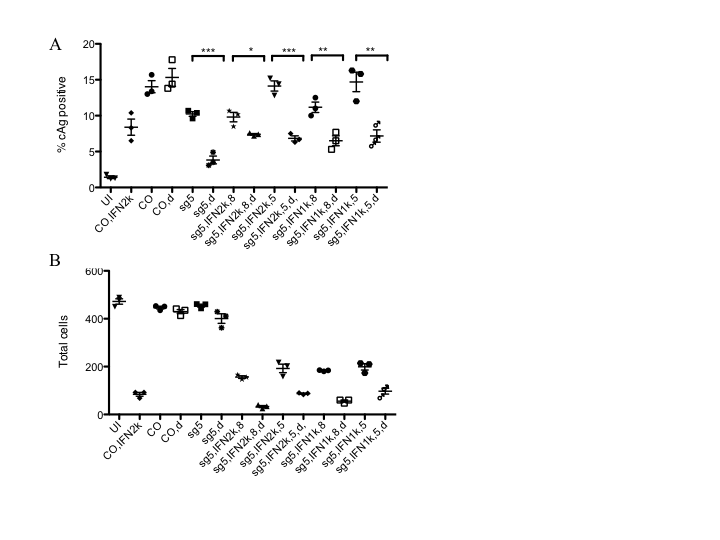

Supplement: Supplementary Figure S3 — Toxicity of IFN-α. [file mtna201468x3.tiff]
