# Supplementary material for: Targeting Hepatitis B Virus With CRISPR/Cas9
Source: Mol Ther Nucleic Acids. 2014 Dec 16;3(12):e216–. doi: 10.1038/mtna.2014.68 (PMC4272409; doi:10.1038/mtna.2014.68)
Supplement: Supplementary Table S1 — Nucleotide sequence of DNA oligomers. [file mtna201468x4.doc]

Table S1

| Name | Sequence* | Pos.** | Assay |
| --- | --- | --- | --- |
|  |  |  |  |
| Sg5 | caccCATTCGGTGGGCGTTCACGG  aaacCCGTGAACGCCCACCGAATG | 3006 | Production of lentivirus |
| Sg6 | caccAATGTCAACGACCGACCTTG  aaacCAAGGTCGGTCGTTGACATT | 3048 | Production of lentivirus |
| Sg7 | caccTTTGAAGTATGCCTCAAGGT  aaacACCTTGAGGCATACTTCAAA | 3081 | Production of lentivirus |
| Sg10 | cacc GCAGAGGTGAAAAAGTTGCA  aaacTGCAACTTTTTCTCCTCTGC | 21 | Production of lentivirus |
| HB2892f | CTCTCTTTACGCGGACTC | 2892 | Surveyor assay |
| HB3148r | GCGCAGACCAATTTATGCC | 3148 | Surveyor assay |
| cccDNAf | GCCTATTGATTGGAAAGTATGT | 2331 | CccDNA-specific PCR |
| cccDNAr | AGCTGAGGCGGTATCTA | 188 | CccDNA-specific PCR |
| rcDNAf | GTTGCCCGTTTGTCCTCTAATTC | 1825 | rcDNA PCR |
| rcDNAr | GGAGGGATACATAGAGGTTCCTTGA | 1924 | rcDNA PCR |
| MitoF*** | TGAGATTAGTAGTATGGGAG | 7901 | Mitochondrial DNA |
| MitoR | CACCCTATTAACCACTCACG | 8215 |  |

* the sequence for the HBV-specific portion of the sgRNAs are indicated in capital letters.

** the position of the 5’ end on the ayw genome where the A of the AUG codon for pre-C is position 1.

*** Primers for PCR amplification of mitochondrial DNA were obtained from Levin et al. .

Figure S1. Nucleotide sequence of NdeI-to-AgeI fragment in pLX-SG1. The fragment contains to two BsmBI/Esp3L sites for cloning of DNA oliogs encoding the sequence specific portion of the selected guide RNA. Restriction sites are underlined in bold face.

Figure S2. HepAD38 cells were infected with lentivirus vectors expressing the respective guide RNAs. Chromosomal DNA was extracted 6 days after infection. HBV DNA was PCR amplified with primers HB2928f and HB3148r flanking the target regions of the three sgRNAs. PCR products were digested with the Surveyor nuclease (Materials and Methods) and electrophoresed through a 2% agarose gel. FL; full length PCR fragment.

Figure S3. Toxicity of IFN-α. NTCP/Cas9 cells were infected with HBV and maintained with or without dox (d, 1 μg/ml). IFN-α (IFN) was added 2 days or 5 days after HBV infection for 5 or 8 days before the cells were processed for IF. The concentration of IFN-α was 2000 IU/ml (2k) or 1000 IU/ml (1k) as indicated. A) Fraction of HBcAg positive (infected) cells. B) Total cell counts.
